# Metal debris concentrations in soft tissues adjacent to loosened femoral stems is higher in uncemented than cemented implants

**DOI:** 10.1186/1471-2474-15-267

**Published:** 2014-08-07

**Authors:** Krzysztof Kmieć, Marek Synder, Piotr Kozłowski, Marek Drobniewski, Marcin Sibiński

**Affiliations:** 1 Clinic of Orthopaedics and Paediatric Orthopaedics, Medical University of Łódź Poland, Drewnowska 75, 91-002 Lodz, Poland

**Keywords:** Metal debris, Stem loosening, Hip revision, Cemented implants, Uncemented implants

## Abstract

**Background:**

There are still many questions related to aseptic femoral stem loosening. Systemic and local immune responses to the implanted “foreign body” is one of the reasons for loosening. The purpose of the study was to measure metal ion concentration (Ti, Co, Cr, Mo, Ni, Al) around loosened femoral stems and compare their levels around uncemented and cemented implants.

**Methods:**

This paper reports 50 hips operated for isolated stem loosening, in 50 patients at the mean age of 57 years (from 21 to 87). There were 25 cemented (Co,Cr_29,_Mo,Ni) and 25 uncemented (Ti, Al) stems. The mean follow-up from primary hip replacement to revision was 10.1 years (from 0.5 to 17). During the procedure, scar tissue around the stem was taken for analysis of metal ions.

**Results:**

The concentrations of titanium and aluminium in soft tissues around uncemented loosened stems were higher than cemented ones (p < 0.001, p < 0.001 respectively). However, no statistically significant differences were observed between both types of stems in terms of ions of the metal of which cemented implants had been made of (Co, Cr, Mo, Ni).

**Conclusions:**

In soft tissue around a loosened stem, the concentrations of metal ions from implants are much higher in case of uncemented stems than of cemented ones. Metal ions from *vitalium* femoral heads were found around uncemented stems in similar values to cemented streams.

## Background

There are still many questions related to aseptic femoral stem loosening which, as a clinical problem, remains till now unresolved. On the other hand, this lack of clear and simple explanation of the mechanisms, which are behind the stem loosening process. There is a complexity of different factors, resulting from the anatomical structures, external to the femoral stem, biased by patient’s body biomechanisms, influenced the way the stem has been implanted, as well as affected by the systemic and local immune responses to the implanted “foreign body” [1-3]. It should also be noted that the materials, which the implants are produced of (metals, polyethylene, ceramics) significantly differ in their physicochemical characteristics from the properties of live tissues, if not infrequently inducing mutual reactions [4]. First of all, the implants are submitted to physical laws of irreversible wear and products of this wear deposit in tissues, where they may trigger various harmful and, by all means, adverse effects [5,6].

Revisions of hip arthroplasties revealed particles of metal wear, both on the surface of removed implants and in surrounding tissues. The aim of the study was to measure metal ion concentration (titanium - Ti, cobalt - Co, chrome - Cr, molybdenum - Mo, nickel - Ni, aluminium - Al) around loosened femoral stems and compare their levels around uncemented and cemented implants.

## Methods

During the years 2007 and 2009, a total of 172 patients were submitted to revision hip arthroplasty, performed at our Department for aseptic cup and/or stem loosening. Out of them, 50 hips were operated for isolated stem loosening, 99 for isolated cup loosening and 23 for loosening of both components. Only homogenous group of patients were included in the study. To eliminate influence of cup loosening on irons metal level, we limited our study to hips with isolated stem loosening. This paper reports 50 hips, operated in 50 patients for aseptic, isolated femoral stem loosening, one of them having, additionally, stem fracture. There were 35 women and 15 men in the group at the mean age of 57 years (the age range from 21 to 87 years). The left hip was operated in 24 and the right one in 26 cases. The indications for primary hip replacement included idiopathic coxarthrosis in 27 hips, femoral fracture in 11, dysplastic coxarthrosis in 9 and aseptic necrosis of femoral head in 3. Different types of hip replacement were performed in the study group. Half of them had cemented implants (Weller’s *(Aesculap)* in 15, Exeter *(Howmedica)* in 5, Centrament *(Aesculap)* in 5 patients), while the other half uncemented stem implants (Mittelmeier *(Osteo)* in 9, Parhofer – Mönch *(Aesculap)* in 8, Bicontact *(Aesculap)* in 8 patients). The cemented stems were made of *vitalium* (Co,Cr_29,_Mo,Ni) and the uncemented ones of titanium, aluminium and vanadium alloys. In all patients, *vitalium* heads were used. All the cemented cups were made of polyethylene. The uncemented cups were made of titanium alloy with a polyethylene insert. No hybrid arthroplasties were performed in the group. The mean follow-up from primary hip replacement to revision was 10.1 years (the range from 6 months to 17 years). There was no statistical differences between cemented and uncemented stem group in terms of: age (53 versus 60 years, respectively, p = 0,2), gender (p = 0,75), follow-up (9.8 versus 10.4 years respectively, p = 0,5) or indication for primary surgery (p = 0,8).

It was a prospective study. The diagnosis of implant loosening was obtained on the basis of clinical examination and radiographic evaluation and finally confirmed during revision hip arthroplasty. Stem loosening was diagnosed on the basis of radiographic criteria proposed by Gruen et al. [7]. Diagnosis of cup loosening was set with the use of DeLee and Gruen classification system [8].

Revision hip arthroplasty was performed in supine position from the anterolateral approach between the *gluteus medius* and the *vastus lateralis* muscle or, in case of extensive soft tissue scaring, the transgluteal approach was applied. Scar tissue and ectopic bone were removed. During the procedure, scar tissue around the stem was taken for analysis of metal ions.

Soft tissue samples, collected during revision operation, were analyzed for ions of the metals from the stems had been (Ti, Co, Cr, Mo, Ni and Al). The measurements were performed by ICP – MS (*Inductively Coupled Plasma Mass Spectrometry*). Calibration of the spectrometer was done, using the *ICP multi – element standard solution VI* by Merc. Before analysis, the probes were processed by microwave energy in solution of an MLS 1200 system by Milestone and of properly concentrated and spectrally clear nitric acid (HNO_3_). The quality of obtained results was verified by control samples.

The research project was approved by the Bioethics Commission at our institution (Protocol No. RNN/2/12/KE). Written informed consent for participation in the study was obtained from participants.

Statistical analysis. The obtained results were submitted to a statistical analysis by the STATISTICA 10.0 PL software. Before an analysis of measurable features was undertaken, the compatibility of their distributions with the normal distribution was evaluated. The λ - Kolmogorov test of goodness of fit with normal distribution was applied. Since the distributions of the analysed features significantly differed from the normal distribution, the Mann–Whitney test was applied for a comparison of medians. For comparison of demographic data between cemented and uncemented group was done using chi^2^ test, ANOVA and Student T test. Values p < 0.05 were regarded statistically significant.

## Results

The concentrations of titanium in soft tissues around uncemented loosened stems were about hundred times higher than in soft tissues around cemented ones. The concentration of aluminium was about thirteen times higher in case of uncemented stems than of cemented ones. Both differences were statistically significant. However, no statistically significant differences were observed between both types of stems in terms of ions of the metal of which cemented implants had been made of (Co, Cr, Mo, Ni) (see Table 1).

**Table 1:** Metal ion concentrations in cemented and uncemented stems, together with statistical comparison

**Metal ions**	**Type of stem fixation**	**Parameters of ion concentration in μg/kg**						**P and Z value**
		**min**	**max**	**x**	**Me**	**SD**	**v (%)**	
Titanium	Cemented	118.6	1196	516.7	423.4	303.2	58.7	**z = 5.937; p < 0.001**
	Uncemented	768.5	999999	55308.6	12268.6	197431.8	356.9	
Aluminium	Cemented	103.5	1234.5	278.8	154.7	270.7	97.1	**z = 5.879; p < 0.001**
	Uncemented	567.8	7977.2	3779.8	3464.7	2374.0	62.8	
Nickel	Cemented	38.8	204230.4	8580.5	365.5	40765.5	475.1	z = 1.572; p > 0.05
	Uncemented	54.4	1353.7	568.3	468.2	438.5	77.2	
Chrome	Cemented	88.6	1494.6	532.3	347.5	451.6	84.8	z = 0.854; p > 0.05
	Uncemented	113.4	1345.8	553.7	455.7	347.8	62.8	
Cobalt	Cemented	2.5	327.6	85.9	23.3	108.6	126.5	z = 1.717; p > 0.05
	Uncemented	11.2	367.3	124.4	44.8	126.3	101.5	
Molybdenum	Cemented	32.9	47855.3	2165.2	198.8	9522.7	439.8	z = 1.455; p > 0.05
	Uncemented	12.9	999999	40787.9	444.7	199841.3	489.9	

Min – minimum, max – maximum, x- mean, Me - median, SD – standard deviation , v (%) – variance, p – statistical significance (P – value), z – z value.

Bold fonts indicate significant differences.

## Discussion

Despite the continued process of improving femoral stem structures and technologies, as well as more and more effective surgical techniques, aseptic loosening of implanted femoral stems has remained one of the major problems of femoral joint arthroplasty. It is generally known that, in *in vivo* conditions, metal implants release ions to surrounding tissues, which ions permeate then with blood to internal organs of operated patient. High concentrations of ions result in numerous. physiologically adverse effects, including cytotoxicity, genotoxicity, carcinogenicity and increased sensitivity (allergy) to metals [3,9,10].

Available reports provide examples of increased concentrations of cobalt, chromium, nickel, titanium, molybdenum, aluminium and vanadium ions in implant surrounding tissues [11,12]. It has also been proven that ions of these metals affect bone metabolism, compromise the immune system, trigger late type allergy and induce pathophysiology of aseptic implant loosening process in operated patients after femoral joint arthroplasty [10]. Additionally, the metal parts of implants undergo direct biocorrosion, caused by the activity of osteoclasts, what leads to release of large amounts of wear particles and metal ions to surrounding tissues [13-15]. Released metal ions reversely stimulate both the immune system and bone metabolism via a number of direct and indirect reactions, leading to an increased osteolytic activity in implant surrounding bone [16-19]. Wear particles may develop biological activity along different cellular pathways. They activate macrophages, foreign body giant cells, as well as fibroblasts of the periprosthetic membrane. These cells induce particle-dependent bone resorption by means of proinflammatory cytokines, such as IL-1beta, TNF-alpha, IL-6 and PGE2. These factors activate osteoclasts, as well as suppress osteoblasts [20].

This article reports ion concentration in a homogenous group of patients, that had isolated femoral stem loosening. In our study, ions of stem metals permeated from uncemeneted implants into surrounding tissues in concentrations higher than for cemented implants. Ions from metals of the evaluated cemented implants were found in tissues surrounding bone cement in concentrations insignificantly different from concentrations of those ions around uncemented stems. Interestingly, metal ions from *vitalium* were found around uncemented stems sometimes in values higher than in streams made of *vitalium*. This observation suggests that these metal debris comes from metal femoral heads and may be a result of electrolysis process between two types of metal.

Similar findings were reported by Hirakawa et al., who analyzed wear debris from failed total hip in 123 tissue samples. More particles were found adjacent to failed titanium-alloy stems and cups than to all-cobalt-chromium-alloy prostheses. Higher concentrations of particles were found in fixations without cement, longer follow-up and in younger patients [21]. In Brien’s report, levels of metal ions from femoral stems were evaluated in the synovial membrane and in the arthral fluid. The applied implants were made of stainless steel, a kobalt-chromium alloy and titanium alloys and fixed in bone with PMMA. Tissues were collected from stable and aseptically loosened implants and their analysis demonstrated that in all the studied groups of stable implants, the levels of metal ions in the arthral fluid were similar. In turn, aseptic implant loosening coexisted with increased levels of metal ions in the arthral fluid and the synovial membrane, where that increase was disproportionately large in case of titanium alloy implants vs. those of steel and the Co-Cr alloy [22]. Similarly Huo, while evaluating a quantitative distribution of metal ions in tissues adjacent to implants and in the articular capsules of patients with aseptic loosening of cemented femoral stem, demonstrated that the levels of Co and Cr ions were, on the average, 4 times higher and Ti ion concentrations were 42 times higher in direct contact with a loosened implant than in the articular capsule [23].

## Conclusions

In soft tissue around a loosened stem, the concentrations of metal ions from implants are much higher in case of uncemented stems than of cemented ones. Metal ions from *vitalium* femoral heads were found around uncemented stems in similar values to cemented streams.

## Competing interests

The authors declare that they have no competing interests.

## Authors’ contributions

MSi, MSy, KK, PK, MD designed the research, acquired the data and revised it critically for important intellectual content. MSi,, KK analyzed the data and performed statistical analyses. MSi, MSy, KK, MD contributed to interpretation of data. MSi, KK, PK drafted the manuscript. All authors read and approved the final manuscript.

## Pre-publication history

The pre-publication history for this paper can be accessed here:

http://www.biomedcentral.com/1471-2474/15/267/prepub
